# How Stress Hinders Health among Chinese Public Sector Employees: The Mediating Role of Emotional Exhaustion and the Moderating Role of Perceived Organizational Support

**DOI:** 10.3390/ijerph16224408

**Published:** 2019-11-11

**Authors:** Yuanjie Bao, Wei Zhong

**Affiliations:** 1 School of Public Administration and Policy, Renmin University of China, Beijing 100872, China; baoyuanjie@ruc.edu.cn; 2 School of Public Policy and Management, Tsinghua University, Beijing 100084, China

**Keywords:** hindrance stressor, emotional exhaustion, perceived organizational support, physical health, mental health

## Abstract

Drawing on the conservation of resources theory, this study examines the detrimental effect of hindrance stressors on self-rated health among a sample of Chinese public sector employees. Analysis of survey data based on 404 MPA students from a leading Chinese university who are working in various public organizations across China suggested that hindrance stressors were negatively related to both physical and mental health (*β* = −0.11, *p* < 0.01 and *β* = −0.38, *p* < 0.001, respectively), and emotional exhaustion mediated those relationships (95% bias-corrected confidence intervals for the indirect effects on physical and mental health based on 5000 bootstrapped samples were −1.64 to −0.35 and −3.51 to −1.81, respectively, excluding 0). Furthermore, perceived organizational support moderated the effect of hindrance stressors on emotional exhaustion (*β* = −0.10, *p* < 0.05), and moderated the indirect effects of hindrance stressors on physical and mental health via emotional exhaustion (index of moderated mediation was 0.116 with bootstrapped confidence interval of 0.018–0.296 for physical health, and 0.317 with bootstrapped confidence interval of 0.008–0.663 for mental health). The effects of hindrance stressors were weaker when perceived organizational support was high, suggesting a moderating effect. Our findings not only provide important theoretical contributions to the literature on public employees’ work-related stress and associated health outcomes, but also offer practical implications to those who are interested in stress intervention to improve the wellbeing of public employees and general society.

## 1. Introduction

Public sector employees work in highly stressful institutional and organizational environments which have profound impacts on their wellbeing and health-related outcomes [1]. However, insufficient research attention has been devoted to understanding the effects of public sector employees’ work stress [2], especially how the stress deteriorates their self-rated health [3]. On the one hand, work-related stress of public sector employees is understudied, with “only a handful of studies” focused on stress in public service agencies [2] (p 89). On the other hand, self-rated health of public sector employees is not recognized as an important outcome variable in public health literature, reflected in the limited number of studies that used health as outcome [1,2,4].

Considering public sector employees’ influences over the general society’s functioning, this research gap is very unfortunate and problematic [5,6,7]. Public sector employees’ stress and health not only influence public sectors’ performance, but also exert profound influences over the general society. Only with a healthy and effective public workforce is it possible to have a prosperous society. Recognizing this important theoretical and practical issue, the current study examines the stress–strain relationship among a sample of Chinese public sector employees. Specifically, drawing on the conservation of resources theory [8,9], this study examines how and when public sector employees’ perceived hindrance stressors at work are related to their self-rated physical and mental health, focusing on the mediating role of emotional exhaustion and the moderating role of perceived organizational support (POS).

By examining the aforementioned relationships, the current study makes two contributions to the ongoing stress and health discussion. First, the stress research in public service contexts is very localized in that it focuses on specific work stressors related to the nature of public institutions, organizations, or jobs. Stressors such as red tape [10], financial stress and job insecurity [11], organizational politics [12], organizational changes related to new public management movement [13], and citizen demand [2] were identified as important stressors within the public service context. However, this localization of the stress concept makes it very hard to compare and synthesize the stress study of public sector employees with the stress study of the general population [1]. This study applies the concept of hindrance stressors within public service contexts [14] and examines whether emotional exhaustion mediates the relationship between hindrance stressors and health outcomes. The health condition of public employees is becoming a concern [15]. For example, it was found that Chinese civil servants’ mental health is worse compared to the general population [16], which echoes the findings from the United Kingdom [17]. Furthermore, medical data of Chinese civil servants showed that their physical health status conditions such as overweight, dyslipidemia, and hepatic lipidosis were cause for alarm [18]. By relating hindrance stressors with both physical and mental health among Chinese public sector employees, this study not only extends the nomological network of stress in the public service context, but also provides new empirical evidence on the relationships between work stressors and health-related outcomes from Eastern cultures [19].

Second, public managers are facing double difficulties when tackling work stress. There might be the coexistence of profound hindrance stressors and restricted extrinsic motivators [20], and the intrinsic motivators in public jobs might not be always sufficient to balance hindrance stressors [21,22]. Researchers are calling for more organizational resources to mitigate the effects of work stressors [23,24,25]. POS is considered as an important form of organizational resource that might complement the resource loss induced by hindrance stressors and buffer the detrimental effects of those stressors [26]. Organizational support commonly provided in the public sector in China could include practices such as time off for education, recognition for contribution, newer technology and work equipment, additional benefits, and family supportive practices. The effects of POS might be especially pronounced in the Chinese public sector, because traditionally the public sector should take care of its employees with good resources and benefits, making them free of worries and full of devotion. To identify possible stress interventions in the public sector, the current study follows the conservation of resources (COR) theory [8] and examines whether public sector employees’ POS would moderate the relationship between hindrance stressors and emotional exhaustion and thus moderate the indirect effects of hindrance stressors on physical and mental health via emotional exhaustion.

## 2. Theoretical Background and Hypotheses Development

### 2.1. Work Stress among Public Sector Employees

Working for public sectors is stressful [27]. Inherent institutional, organizational, and professional characteristics can induce profound work-related stress among public sector employees [28]. Although not as abundant as in the business administration and psychology research [1], there is growing research examining the nature, antecedent, outcome, and effect mechanism of work stress within the public sector [1,2,29]. Various work stressors specific to public sectors are identified, including low wages and limited job control [17], job insecurity [30], workplace politics [12], organizational change [13], work overload [29], civil service reform [31], red tape [10], role conflict [32], goal ambiguity [33], emotional requirement [34,35], and citizen demand [2]. It was found that these work stressors were detrimental to employees’ attitudinal outcomes, such as burnout [36,37], job satisfaction [28,29,38], and turnover intention [39,40].

Although growing and promising, the stress research targeting public sector employees is only in its infancy. Particularly, although public sector employees’ health conditions have been increasingly raising concerns [17,30,41], limited empirical studies have comprehensively examined the relationships between work stressors and their health. For example, civil servants’ mental health is a neglected subject with only two exceptions [1,42]. Thus, the current study draws on the COR theory to examine whether, how, and when hindrance stressors would be detrimental to Chinese public employees’ self-rated health.

### 2.2. Hindrance Stressors, Emotional Exhaustion, and Health

Hindrance stressors are defined as “work-related demands or circumstances that tend to constrain or interfere with an individual’s work achievement” [43] (p 68). They are considered detrimental to employees’ attitudinal, behavioral, and health-related outcomes [14,44,45]. This study chooses to focus on hindrance stressors mainly because they constitute an overarching concept that includes perceived organizational politics, bureaucratic procedure, red tape, and role ambiguity, which characterize typical deleterious work requirements that public sector employees face [14].

According to the COR theory, individuals “strive to retain, protect, and build resources and that what is threatening to them is the potential or actual loss of these valued resources” [8] (p 516). Individuals experience stress when they think their valued resources are at risk of being deprived [9,46]. When confronted with a demanding and stressful work environment that requires individuals to invest valuable personal resources, individuals would feel the deprivation of resources. This perceived loss could lead to job strain [14] and further induce health-related problems such as headache and body pain [47].

Coping with hindrance stressors requires tremendous resource investment from employees, which leads to strain reactions. It was found that hindrance stressors can cause cognitive and emotional strain [48], raise anxiety and anger [49], and have negative effects on affective state [50]. These conditions could then lead to health problems [51]. It was also found that hindrance stressors might have negative effects on individuals’ self-concepts including self-esteem [47] and self-efficacy [52]. Because self-concepts are related to wellbeing [53] and depression [54], it could be expected that hindrance stressors are negatively related to physical and mental health through deteriorating self-concepts. In this vein, Kim and Beehr [47] found that hindrance stressors are related to ill health through the mediating effect of organization-based self-esteem. Furthermore, under the influence of hindrance stressors, individuals might feel as though they are losing control at work such that they consider job goals as either unclear or impossible to achieve [55]. They might resort to maladaptive coping styles that could hurt their emotional wellbeing and eventually their health [14]. It was reported that a high level of stress is related to rumination and then to lower sleep quality and less restoration activities [56]. While rumination, sleep quality, and restoration are related to health, in this regard, hindrance stressors would lead to health problems. Based on these theoretical arguments and empirical evidence, the following hypotheses are proposed:

**Hypothesis** **1.** *Hindrance stressors are negatively related to public sector employees’ self-reported physical and mental health.*

The two-dimensional stressor framework underlying the conception of hindrance stressors posits that hindrance stressors cause both proximal and distal outcomes [14,57]. Also, according to the COR theory, resource loss induced by stressors leads to psychological strain [58], such as emotional exhaustion, and then to more distal outcomes. Following these propositions, this study examines the mediating role of emotional exhaustion in the stressor–health relationship.

Emotional exhaustion is “a type of strain that results from workplace stressor” [59] (p 160). According to the job demands-resources (JD-R) model, it occurs when job demands exceed employees’ capacity [60,61]. Hindrance stressors encompass unmanageable job demands that are out of personal control, which exceed employees’ coping abilities and foster emotional exhaustion [60]. There is also ample evidence relating hindrance stressors to emotional exhaustion [58,62,63]. Meanwhile, individual health could be deteriorated by emotional exhaustion [64]. Previous research found that emotional exhaustion exerted negative effects on both physical and mental health [65,66,67,68]. It is therefore proposed that:

**Hypothesis** **2.** *Emotional exhaustion partially mediates the relationship between hindrance stressors and self-reported physical and mental health.*

### 2.3. The Moderating Role of POS

The COR theory claims that individuals can rely on other resources to compensate the resources lost [69,70]. That is to say, when individuals are facing negative effects due to the loss of resources from one source, the negative effects can be attenuated by resources from another source [46]. Additional resources can come either from personal psychological strength or from organizational environment [70]. POS is an important form of organizational resources. In public sectors, employees may not have resources such as extrinsic rewards [20] or supervisory support [71]. The importance of POS in this case becomes more salient. This study investigates its moderating role in the relationships between hindrance stressors, emotional exhaustion, and self-rated health. We claim that for public employees, the provision of resources from POS would be particularly pronounced.

POS is employees’ perception that their organizations appreciate their contributions, care about their well-being, and treat them nicely [26]. The COR theory posits that POS “provides additional resources for employees to more effectively deal with work stress” [55] (p 254). Empirically, it was found that POS moderated work stressors’ effects on organizational citizenship behavior by providing additional resources [72]. POS also moderated the effects of role conflict on emotional exhaustion [73], and it reduced the stressful impact on employees’ negative mood [74]. In another study [75], POS and hindrance stressors interacted to predict employee creativity. Synthesizing the above arguments and empirical findings, the following hypothesis is proposed:

**Hypothesis** **3.** *POS moderates the relationship between hindrance stressors and emotional exhaustion, such that the positive relationship will be weaker when POS is high.*

Hypotheses 1 and 2 suggest an indirect effect of hindrance stressors on both physical and mental health via emotional exhaustion, and Hypothesis 3 suggests that POS moderates the effect of hindrance stressors on emotional exhaustion. Considering that POS is an important antecedent of health-related outcomes [76,77], it is logical to integrate these three hypotheses into a moderated mediation model [78,79], in that the aforementioned indirect effects also depend upon POS. It is hypothesized that:

**Hypothesis** **4.** *POS moderates the indirect effect of hindrance stressors on self-reported physical and mental health through emotional exhaustion, such that the indirect effect is weaker when POS is high.*

**Figure 1:**
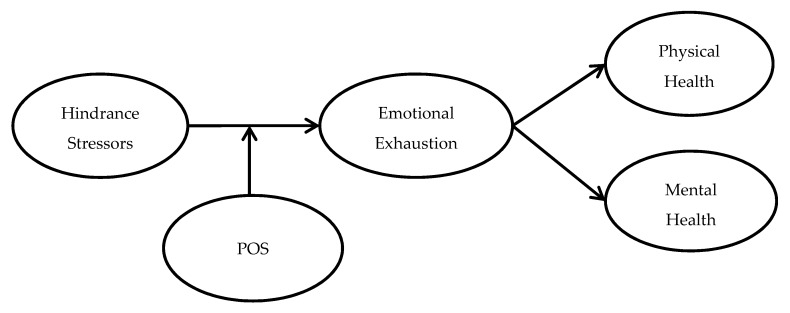
Hypothesized model. POS = perceived organizational support.

The proposed theoretical framework is depicted in Figure 1.

## 3. Methods

### 3.1. Sample and Procedures

The target population of this study was the incumbent employees in Chinese public sectors. Considering the difficulties in surveying Chinese public sector employees [1], this study adopted a convenient sampling approach by surveying the Master of Public Administration (MPA) students from a university located in Beijing. This program is among the first group of MPA programs established in China. It is offered to those who have more than three years of working experience. Its students are public sector employees throughout China.

A total of 700 invitation emails including an online survey link were sent to all MPA students in the university. Participants received a link in their emails and could answer the survey using their personal computers or smartphones. The survey contained 31 items measuring the studied variables and controls. The approximate length of time to complete the survey was 10 to 15 min. The academic purpose of the survey was introduced at the beginning of the survey, and anonymity and confidentiality were guaranteed. A total of 419 respondents returned their questionnaires, at a response rate of 59.9%. After excluding 15 questionnaires that either had missing data or were not filled by public sector employees, the final sample size was 404. Among these 404 respondents, 243 (60.1%) were female. The average age was 30.3 years (SD = 4.49), and the average organizational tenure was 5.22 years (SD = 3.89).

### 3.2. Measures

Variables were measured with established scales with good psychometrical properties [26,43,80,81]. This study used translated Chinese versions of these scales because they have been successfully used to adapt to the Chinese context in previous studies.

#### 3.2.1. Hindrance Stressors

Perceived hindrance stressors were measured using five items [43]. Each item was scored on a five-point Likert scale to indicate the level of stress associated with a stressor. The scale ranged from 1, representing “no stress”, to 5, representing “a great deal of stress”. A sample item was “The amount of red tape I need to go through to get my job done”. The Cronbach’s alpha was 0.78.

#### 3.2.2. Emotional Exhaustion

Five items from the Maslach Burnout Inventory General Survey (MBI-GS) [80] were used to measure emotional exhaustion. A sample item was “Working all day is really a strain for me”. Responses were recorded on a seven-point frequency of occurrence ranging from 0, representing “never”, to 6, representing “always”. The Cronbach’s alpha was 0.92.

#### 3.2.3. POS 

Six items were used to measure POS [26]. A sample item was “My organization cares about my objectives and values”. Responses were recorded on a seven-point Likert scale ranging from 1, representing “strongly disagree”, to 7, representing “strongly agree”. Based on results from confirmatory factor analysis (CFA) reported below, the sixth item, “If given the opportunity, the organization would take advantage of me”, which was reversely coded, loaded poorly compared to other items. This item was deleted for further analyses. The Cronbach’s alpha for the remaining five items was 0.90.

#### 3.2.4. Physical and Mental Health

Self-rated health was measured using 12-item Short Form Health Survey (SF-12) of the health-related quality of life (HRQOL) scale [81]. Physical health was measured with six items such as “Accomplishing less than one would like as a result of physical health”, and mental health was measured with six items such as “Feeling down-hearted and blue”. Responses to items were recorded and computed based on the standard scoring and algorithm of this scale [82]. Each score ranges from 0 (the worst possible health state) to 100 (the best possible health state). Higher scores for physical and mental health indicate better function and subjective feelings. The Cronbach’s alphas for physical and mental health were 0.72 and 0.76, respectively.

#### 3.2.5. Control Variables

Respondents’ gender (0 = female, 1 = male), age (in years), and organizational tenure (in years) were included as control variables, as these variables might be related to emotional exhaustion and self-rated health outcomes [1,4,15,19,60,61].

### 3.3. Analytical Techniques

Confirmatory factor analysis (CFA) was used to test the convergent and discriminant validity of examined variables. Then, hierarchical regression analysis was used to test the hypotheses. Specifically, both Sobel test and the bootstrapping approach [79,83] were used to test the mediation. The moderated mediation hypothesis was tested using the PROCESS macro designed by Hayes [79].

## 4. Results

### 4.1. Measurement Model

Prior to hypothesis testing, a series of CFA were performed to test the convergent and discriminant validity of study variables, including hindrance stressors, emotional exhaustion, and POS (physical and mental health were not included in these analyses because of their scoring systems). As shown in Table 1, the hypothesized three-factor model (hindrance stressors, emotional exhaustion, and POS) fitted data well (*χ*^2^ (87) = 212.35, *χ*^2^/df = 2.44, *p* < 0.01; RMSEA = 0.06 (95 CI = 0.05–0.07, *p*-close = 0.06), SRMR = 0.04, CFI = 0.96, TLI = 0.96). The factor loadings of the items were all above 0.50 (ranging from 0.59 to 0.86) and significant at 0.001 level.

This three-factor model was also compared with four alternative measurement models. The chi-squared difference tests in Table 1 show that the three-factor model fitted best with the data. Furthermore, for the three-factor model, the composite reliability scores were all above 0.7 (ranging from 0.79 to 0.92), the maximum shared variance scores were smaller than the average variance extracted scores, and the square root of the average variance extracted scores were greater than the inter-contrast correlations [84,85]. These results suggest good validity.

### 4.2. Descriptive Statistics and Correlations

The means, standard deviations, and correlation coefficients among variables are reported in Table 2. Hindrance stressors were positively related to emotional exhaustion (*r* = 0.52, *p* < 0.01), and negatively related to physical health (*r* = −0.11, *p* < 0.05) and mental health (*r* = −0.37, *p* < 0.01). Emotional exhaustion was negatively related to POS (*r* = −0.29, *p* < 0.01), physical health (*r* = −0.18, *p* < 0.01), and mental health (*r* = −0.50, *p* < 0.01). POS was positively related to physical health (*r* = 0.11, *p* < 0.05) and mental health (*r* = 0.30, *p* < 0.01). Physical and mental health were not related to each other. These results are consistent with the research hypotheses.

### 4.3. Hypothesis Testing

Hypothesis 1 suggests that hindrance stressors are negatively related to physical and mental health. As shown in Table 3 (Models 6 and 9), after controlling for the effects of gender, age, and organizational tenure, hindrance stressors are negatively related to physical health (*β* = −0.11, *p* = 0.01) and mental health (*β* = −0.38, *p* < 0.001), supporting Hypothesis 1.

Hypothesis 2 suggests that emotional exhaustion partially mediates the effects of hindrance stressors on physical and mental health. Model 2 in Table 3 reveals that hindrance stressors are positively related to emotional exhaustion (*β* = 0.53, *p* < 0.001). Models 7 and 10 indicate that when hindrance stressors and emotional exhaustion were entered simultaneously, emotional exhaustion was negatively related to physical health (*β* = −0.20, *p* < 0.01) and mental health (*β* = −0.40, *p* < 0.001), which suggests the mediation effect of emotional exhaustion [86]. Sobel test and bootstrapping were used to test the significance of these indirect effects. For physical health, the Sobel value was −0.95 (SE = 0.29). The z sore was −3.22 (*p* < 0.01), and the 95% bias-corrected confidence interval for the indirect effect based on a 5000 bootstrapped sample was −1.64 to −0.35, excluding 0. The direct effect was −1.12 to 1.00, including 0. Thus, emotional exhaustion fully mediated the relationship between hindrance stressors and physical health. The ratio of indirect to total effect was 0.94. For mental health, the Sobel value was −2.59 (SE = 0.39). The z sore was −6.67 (*p* < 0.001), and the 95% bias-corrected confidence interval for the indirect effect based on a 5000 bootstrapped sample was −3.51 to −1.81, excluding 0. The direct effect was −3.19 to −0.76, excluding 0. Thus, emotional exhaustion partially mediated the relationship between hindrance stressors and mental health. The ratio of indirect to total effect was 0.57. Hypothesis 2 is thus partially supported.

**Figure 2:**
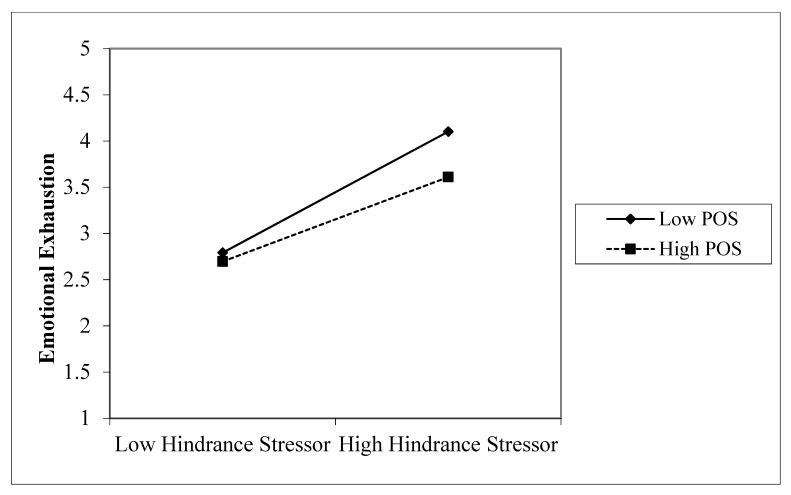
Interactive effects of hindrance stressors and POS on emotional exhaustion. POS = perceived organizational support.

Hypothesis 3 suggests that POS moderates the relationship between hindrance stressors and emotional exhaustion. As displayed in Table 3 (Model 4), when the interaction term between hindrance stressors and POS was entered to predict emotional exhaustion, controlling for the effects of hindrance stressors and POS, the interaction term was significantly and negatively related to emotional exhaustion (*β* = −0.10, *p* < 0.05). Hypothesis 3 is supported. To confirm the direction of this interaction effect, simple slopes were plotted at one standard deviation above and below the mean of POS in Figure 2. As displayed, the slope of the relationship between hindrance stressors and emotional exhaustion was steeper when POS was low (simple slope = 0.655, *t* = 10.190, *p* < 0.001), whereas the slope was flatter when POS was high (simple slope = 0.457, *t* = 5.966, *p* < 0.001).

Hypothesis 4 suggests that the indirect effects of hindrance stressors on physical and mental health via emotional exhaustion are contingent upon different levels of POS. To test this hypothesis, the PROCESS macro was used [83]. For physical health, there was a moderated mediation (0.116 with bootstrapped confidence interval of 0.018–0.296). As shown in Table 4, the indirect effect was conditional on different POS levels: stronger (−0.987, with bootstrapped confidence interval of −1.713 to −0.389) when POS was low, and weaker (−0.688, with bootstrapped confidence interval of −1.323 to −0.232) when POS was high. For mental health, there was a moderated mediation (0.317 with bootstrapped confidence interval of 0.008–0.663). Table 4 reveals that the indirect effect was conditional on different POS levels: stronger (−2.700, with bootstrapped confidence interval of −3.723 to −1.832) when POS was low, and weaker (−1.881, with bootstrapped confidence interval of −2.760 to −1.146) when POS was high. Hypothesis 4 is supported.

## 5. Discussion

### 5.1. Theoretical Implications

The findings of the current study fill some research gaps in the literature and provide implications for future studies. First, the stress–strain relationship is examined among public sector employees. Although stress and associated health problems are raising alarm in the public sector, few empirical efforts have been made link stress with health-related outcomes in this context [1,3,4]. This study provides empirical evidence on the detrimental effects of hindrance stressors on public sector employees’ self-rated health. From the stress perspective, the study identifies one important antecedent of public sector employees’ health status.

Meanwhile, the concept of hindrance stressors has rarely been used in the public service context. Public sector jobs are normally characterized by the prevalence of hindrance stressors, such as organizational politics [12], red tape [10], and role ambiguity [87]. This study validates the measurement and applicability of hindrance stressors in the public sector. It responds to the call of examining the effects of hindrance stressors on health-related outcomes [55]. Further studies are encouraged to use overcharging stress concepts in public service contexts.

Second, consistent with the propositions of the COR theory and JD-R model, this study finds that emotional exhaustion serves as the mediator between hindrance stressors and health-related outcomes. Emotional exhaustion is the most studied dimension of job burnout [59] and is considered as the first developmental stage of burnout [64]. By identifying a key antecedent of emotional exhaustion, this study provides insights on how the burnout of public sector employees develops. Given the increasing interest to study public employees’ burnout [65,88] and engagement [89,90,91], our work offers new insight on the nomological network of burnout and possibly engagement [65,88] in the public sector. This study also provides support for the usage of the COR theory and JD-R model in the public service context [88]. Future work is encouraged to employ these theoretical perspectives to understand public sector employees’ job attitude and behaviors.

Third, this study reveals the moderating effects of POS on the stressor–strain relationship. POS is expected to help public sector employees cope with stressful working environment. However, Jain and her colleagues [72] cautioned that the moderation effect of POS might not be generalized to the public service context. Findings of the current study alleviate this concern. Future studies could explore the moderating effects of other variables, such as public service motivation [1], self-efficacy [6], and supervisory support [92]. Such efforts help answer the challenging and important question of how to recharge public sector employees’ resource loss due to stress.

Furthermore, the current findings on POS’s moderating effects are inconsistent with results from previous studies. For example, POS failed to moderate the relationship between hindrance stressors and role-based job performance [55] and the relationship between hindrance stressors and cognitive or emotional strain [48]. It is possible that the moderating effects of POS might be more pronounced among public sector employees. Future work could examine whether public sector employees consider POS more important than their private counterparts.

Although we find that hindrance stressors relate to health outcomes through emotional exhaustion, some physiological reasons underlying the process through which stress relates to health should be examined further. Possible reasons such as rumination, sleep quality, and lack of restoration should be examined.

### 5.2. Practical Implications

Public managers who are concerned with stress intervention and health promotion can benefit from the findings of this study. First, the profound and detrimental influences of hindrance stressors on both physical and mental health illustrate the alarming influences of organizational politics, red tape, and role ambiguity on public sector employees. Public sectors should pay detailed attention to how organizational procedures, processes, and practices might be perceived by employees. Meanwhile, systemic changes in the government system are needed to reduce such hindrance stressors. Programs proposed by the majority of prior research or implemented in practices to reduce hindrance stressors commonly focus on these stressors per se. They may not be fruitful, since hindrance stressors are out of personal and organizational control. Second, hindrance stressors are related to health-related outcomes through emotional exhaustion. Public sectors could develop strategies to reduce or prevent emotional exhaustion and improve employees’ health conditions. Third, this study reveals that POS can partially mitigate the effects of hindrance stressors. Public sectors could build a supportive work environment and provide additional resources to their employees. Besides, as part of the overall organizational support, support from colleagues could provide socio-emotional support [55].

### 5.3. Limitations and Future Research

There are several limitations in this study. First, the cross-sectional nature of the data of this study does not permit causal claims among variables. Although the proposed model is well grounded in theory and consistent with previous studies, future studies are needed for longitudinal or experimental research to examine the causal relationships among model variables. Although contrary to the theoretical propositions in this study, some might argue that the relationship between health and stress perception might be bidirectional, such that poor health would lead to further stress-coping problems and thus lead to higher levels of perceived stress. Future studies should examine whether there is reverse causation among our studied variables. Second, the convenient sampling procedure might limit the generalizability of the findings to a larger population. Also, since all the participants were recruited from an MPA program, these participants reflect a highly educated pool of public sector employees, which might limit the generalizability of the finding. Furthermore, the appraisal of and response to hindrance stressors might be influenced by personal and cultural factors [5]. Future work could explore whether the relationships examined in this study can also be found in other contexts. In addition, the size of the organizations in which the participants were working was not measured in this study. It is possible that the nature of the perceived hindrance stressors and POS would vary based on the size of the organization. Thus, future work can investigate the effects of organizational characteristics such as organizational size in the process of stress perception. Third, this study targets hindrance stressors because they are more salient in public sectors. However, some researchers argued that hindrance stressors should be examined together with other types of work stressors, such as challenge stressors [45]. Future work could examine both types of work stressors in the same study.

## 6. Conclusions

Previous efforts devoted to examining the relationship between work stress and health-related outcomes among the public service workforce have been limited. Using an overcharging stressor concept and drawing on the COR theory, the current study attempts to address this critical issue, with a specific focus on the mediating role of emotional exhaustion and the moderating role of POS. Findings of the current study extend the understanding of the stressor–health relationship in the public service context, particularly the underlying mechanism and boundary conditions of work stressors’ effects. These findings also provide practical management implications and identify interesting future research directions. Work stress and health issues have become an increasing concern for employees and management, particularly in public sectors, which deserves more systematic investigation from both research and practices.

## Figures and Tables

**Table 1:** Confirmatory factor analysis (CFA) results.

Model	*χ* ^2^	df	*χ*^2^/df	Δ*χ*^2^(Δdf)	RMSEA	SRMR	CFI	TLI
Three-factor model	212.35	87	2.44	---	0.06	0.04	0.96	0.96
Two-factor model 1 ^1^	693.38	89	7.79	481.03 (2) ***	0.13	0.16	0.82	0.79
Two-factor model 2 ^2^	480.16	89	5.40	267.81 (2) ***	0.10	0.09	0.88	0.86
Two-factor model 3 ^3^	1276.19	89	14.34	1063.84 (2) ***	0.18	0.18	0.65	0.59
One-factor model ^4^	1515.34	90	16.84	1302.99 (3) ***	0.20	0.19	0.59	0.51

Δ*χ*^2^ and Δdf denote differences between the three-factor model and other models. RMSEA = root-mean-square error of approximation, SRMR = standardized root-mean-square residual, CFI = comparative fit index, TLI = Tucker-Lewis index. *** *p* < 0.001. ^1^ This model combines hindrance stressors and POS (perceived organizational support) into one factor. ^2^ This model combines hindrance stressors and emotional exhaustion into one factor. ^3^ This model combines POS and emotional exhaustion into one factor. ^4^ This model combines hindrance stressors, POS, and emotional exhaustion into one factor.

**Table 2:** Means, standard deviations, and correlations among variables.

	Mean	SD	1	2	3	4	5	6	7	8
1. Gender	0.40	0.49								
2. Age	30.29	4.49	0.12 *							
3. Organizational Tenure	5.22	3.89	0.08	0.70 **						
4. Hindrance Stressors	2.56	0.79	0.12 *	0.01	0.05	(0.78)				
5. Emotional Exhaustion	2.15	1.20	0.00	−0.14 **	−0.06	0.52 **	(0.92)			
6. POS	4.11	1.29	−0.11 *	0.09	0.06	−0.33 **	−0.29 **	(0.90)		
7. Physical Health	51.66	7.45	0.02	−0.16 **	−0.21 **	−0.11 *	−0.18 **	0.11 *	(0.72)	
8. Mental Health	44.08	9.66	−0.03	0.16 **	0.11 *	−0.37 **	−0.50 **	0.30 **	−0.04	(0.76)

*N* = 404. Cronbach’s alphas are reported in parentheses. POS = perceived organizational support. ** *p* < 0.01, * *p* < 0.05.

**Table 3:** Regression results for mediation and moderation.

Variables	Emotional Exhaustion				Physical Health			Mental Health		
	Model 1	Model 2	Model 3	Model 4	Model 5	Model 6	Model 7	Model 8	Model 9	Model 10
Step 1: Control variables										
Gender	0.02	−0.05	−0.06	−0.06	0.04	0.05	0.04	−0.05	−0.01	−0.03
Age	−0.20 **	−0.16 **	−0.15 *	−0.15 *	−0.02	−0.03	−0.06	0.17 *	0.14 *	0.08
Organizational Tenure	0.08	0.03	0.04	0.04	−0.20 **	−0.20 **	−0.19 **	0.00	0.03	0.05
Step 2: Main effect										
Hindrance Stressors		0.53 ***	0.49 ***	0.46 ***		−0.11 **	−0.01		−0.38 ***	−0.16 **
Step 3: Mediating effect										
Emotional Exhaustion							−0.20 **			−0.40 ***
Step 4: Moderating effect										
POS			−0.12 **	−0.12 **						
Hindrance Stressors × POS				−0.10 *						
Overall F	3.084 *	41.29 ***	35.03 ***	30.51 ***	6.62 ***	6.19 ***	7.32 ***	3.87 **	19.60 ***	30.80 ***
R^2^	0.02	0.29	0.31	0.32	0.05	0.06	0.08	0.03	0.16	0.28
ΔF		152.42 ***	7.37 **	5.78 *		4.72 **	11.20 **		64.94 ***	63.33 ***
ΔR^2^		0.27	0.01	0.01		0.01	0.03		0.14	0.12

*N* = 404. Standardized coefficients are reported. POS = perceived organizational support. *** *p* < 0.001, ** *p* < 0.01, * *p* < 0.05.

**Table 4:** Moderated mediation results for self-rated health across levels of POS.

		Physical Health			
	POS	Conditional Indirect Effect	SE	LL 95% CI	UL 95% CI
Hindrance Stressors	Low (−1.293)	−0.987	0.338	−1.713	−0.389
	High (1.293)	−0.688	0.272	−1.323	−0.232
		Mental Health			
	POS	Conditional Indirect Effect	SE	LL 95% CI	UL 95% CI
Hindrance Stressors	Low (−1.293)	−2.700	0.483	−3.723	−1.832
	High (1.293)	−1.881	0.413	−2.760	−1.146

*N* = 404. Bootstrap sample size = 5000. POS = perceived organizational support. LL = lower limit, CI = confidence interval, UL = upper limit.
